# Rare Protein-Truncating Variants in *APOB*, Lower Low-Density Lipoprotein Cholesterol, and Protection Against Coronary Heart Disease

**DOI:** 10.1161/CIRCGEN.118.002376

**Published:** 2019-05-21

**Authors:** Gina M. Peloso, Akihiro Nomura, Amit V. Khera, Mark Chaffin, Hong-Hee Won, Diego Ardissino, John Danesh, Heribert Schunkert, James G. Wilson, Nilesh Samani, Jeanette Erdmann, Ruth McPherson, Hugh Watkins, Danish Saleheen, Shane McCarthy, Tanya M. Teslovich, Joseph B. Leader, H. Lester Kirchner, Jaume Marrugat, Atsushi Nohara, Masa-aki Kawashiri, Hayato Tada, Frederick E. Dewey, David J. Carey, Aris Baras, Sekar Kathiresan

**Affiliations:** 1 Department of Biostatistics, Boston University School of Public Health, MA (G.M.P.).; 2 Department of Cardiovascular and Internal Medicine, Kanazawa University Graduate School of Medicine, Kanazawa, Japan (A. Nomura, A. Nohara, M.K., H.T.).; 3 Program in Medical and Population Genetics, Broad Institute, Cambridge, MA (A.V.K., M.C., S.K.).; 4 Cardiovascular Research Center (A.V.K., S.K.), Center for Genomic Medicine (A.V.K., S.K.), and Department of Medicine (A.V.K., S.K.), Massachusetts General Hospital, Boston, MA.; 5 Samsung Advanced Institute for Health Sciences and Technology, Sungkyunkwan University, Samsung Medical Center, Seoul, Republic of Korea (H.-H.W.).; 6 Cardiology, Azienda Ospedaliero-Universitaria di Parma, University of Parma, Parma, Italy (D.A.).; 7 ASTC: Associazione per lo Studio Della Trombosi in Cardiologia, Pavia, Italy (D.A.).; 8 MRC/BHF Cardiovascular Epidemiology Unit, Department of Public Health and Primary Care (J.D.), University of Cambridge, Cambridge, United Kingdom.; 9 The National Institute for Health Research Blood and Transplant Research Unit (NIHR BTRU) in Donor Health & Genomics (J.D.), University of Cambridge, Cambridge, United Kingdom.; 10 Wellcome Trust Sanger Institute, Genome Campus, Hinxton, United Kingdom (J.D.).; 11 Deutsches Herzzentrum München, Technische Universität München, Deutsches Zentrum für Herz-Kreislauf-Forschung, München, Germany (H.S.).; 12 Department of Physiology and Biophysics, University of Mississippi Medical Center, Jackson, MS (J.G.W.).; 13 Department of Cardiovascular Sciences, University of Leicester, Leicester, United Kingdom (N.S.).; 14 NIHR Leicester Biomedical Research Center, Glenfield Hospital, Leicester, United Kingdom (N.S.).; 15 Institute for Integrative and Experimental Genomics, University of Lübeck, Germany (J.E.).; 16 University of Ottawa Heart Institute, Ottawa, Canada (R.M.).; 17 Cardiovascular Medicine, Radcliffe Department of Medicine and Wellcome Trust Center for Human Genetics, University of Oxford, Oxford, United Kingdom (H.W.).; 18 Department of Biostatistics and Epidemiology, Perelman School of Medicine, University of Pennsylvania, Philadelphia, PA (D.S.).; 19 Regeneron Genetics Center, Tarrytown, NY (S.M., T.M.T., F.E.D., A.B.).; 20 Geisinger Health System, Danville, PA (J.B.L., H.L.K., D.J.C.).; 21 Registre Gironí del Cor group, IMIM (Hospital del Mar Research Institute), Barcelona, Spain (J.M.). CIBER Enfermedades Cardiovasculares (CIBERCV), Barcelona, Spain (J.M.).

**Keywords:** cholesterol, genetics, human, hypobetalipoproteinemia, triglycerides

## Abstract

**Background:**

Familial hypobetalipoproteinemia is a genetic disorder caused by rare protein-truncating variants (PTV) in the gene encoding *APOB* (apolipoprotein B), the major protein component of LDL (low-density lipoprotein) and triglyceride-rich lipoprotein particles. Whether heterozygous *APOB* deficiency is associated with decreased risk for coronary heart disease (CHD) is uncertain. We combined family-based and large scale gene-sequencing to characterize the association of rare PTVs in *APOB* with circulating LDL-C (LDL cholesterol), triglycerides, and risk for CHD.

**Methods:**

We sequenced the *APOB* gene in 29 Japanese hypobetalipoproteinemia families, as well as 57 973 individuals derived from 12 CHD case-control studies—18 442 with early-onset CHD and 39 531 controls. We defined PTVs as variants that lead to a premature stop, disrupt canonical splice-sites, or lead to insertions/deletions that shift reading frame. We tested the association of rare *APOB* PTV carrier status with blood lipid levels and CHD.

**Results:**

Among 29 familial hypobetalipoproteinemia families, 8 families harbored *APOB* PTVs. Carrying 1 *APOB* PTV was associated with 55 mg/dL lower LDL-C (*P*=3×10^-5^) and 53% lower triglyceride level (*P*=2×10^-4^). Among 12 case-control studies, an *APOB* PTV was present in 0.038% of CHD cases as compared to 0.092% of controls. *APOB* PTV carrier status was associated with a 43 mg/dL lower LDL-C (*P*=2×10^-7^), a 30% decrease in triglycerides (*P*=5×10^-4^), and a 72% lower risk for CHD (odds ratio, 0.28; 95% CI, 0.12–0.64; *P*=0.002).

**Conclusions:**

Rare PTV mutations in *APOB* which are associated with lower LDL-C and reduced triglycerides also confer protection against CHD.

## Abstract 2

Supplemental Digital Content is available in the text.

APOB (apolipoprotein B) is a structural component of lipoproteins with a functional role as a ligand that binds to cell-surface receptors, including the LDL (low-density lipoprotein) receptor.^1^ Rare protein-truncating variants (PTVs) that truncate *APOB* lead to familial hypobetalipoproteinemia (FHBL, OMIM no. 107730), an autosomal dominant genetic disorder characterized by low levels of plasma LDL-C (LDL cholesterol).^2,3^ Those affected by FHBL display not only lower LDL-C but also nonalcoholic fatty liver disease.

Mipomersen is an antisense drug approved by the US Food and Drug Administration that targets the mRNA for *APOB* and inhibits the synthesis of the apoB protein. Mipomersen is approved to lower cholesterol in individuals with homozygous familial hypercholesterolemia.^4^ Mipomersen leads to a significant decrease in LDL-C levels in individuals with homozygous familial hypercholesterolemia; however, similar to *APOB* PTVs, mipomersen also leads to fatty liver and elevated liver function test abnormalities.^5^

Carriers of PTVs in *APOB* display lower LDL-C^6^ and triglyceride levels and as such, might be expected to have reduced risk for coronary heart disease (CHD). However, to date, there is little evidence as to whether loss of *APOB* function will affect CHD risk,^7,8^ and a pharmacological test of this hypothesis with mipomersen seems unlikely because of the adverse effects of this therapy. As such, here, we took a human genetics approach to address the following: (1) the extent to which *APOB* PTV carrier status is associated with serum lipid levels using 29 Japanese FHBL families; and (2) whether PTVs in the *APOB* gene are associated with lipid levels and CHD among ≈58 000 individuals from large case-control studies.

## Methods

All participants in the study provided written informed consent for genetic studies. The institutional review boards at the Broad Institute and each participating institution approved the study protocol. To minimize the possibility of unintentionally sharing information that can be used to reidentify private information, a subset of the data generated for this study are available at dbGaP (The database of Genotypes and Phenotypes) and can be accessed at through dbGaP Study Accessions: phs000814.v1.p1 (ATVB [Italian Atherosclerosis, Thrombosis, and Vascular Biology]), phs001398.v1.p1 (BRAVE [Bangladesh Risk of Acute Vascular Events study]), phs000279.v2.p1 (EOMI [Exome Sequencing Project Early-Onset Myocardial Infarction]), phs001098.v1.p1 (JHS [Jackson Heart Study]), phs001000.v1.p1 (Leicester [Leicester Myocardial Infarction]), phs000990.v1.p1 (North German MI [North German Myocardial Infarction]), phs000916.v1.p1 (South German MI [South German Myocardial Infarction]), phs000806.v1.p1 (OHS [Ottawa Heart Study]), phs000883.v1.p1 (PROCARDIS [Precocious Coronary Artery Disease]), phs000917.v1.p1 (PROMIS [Pakistan Risk of Myocardial Infarction Study]), phs000902.v1.p1(Regicor [Registre Gironí del COR (Gerona Heart Registry)]).

The full methods are available in the Data Supplement.

## Results

### Hypobetalipoproteinemia Families

**Figure 1.**
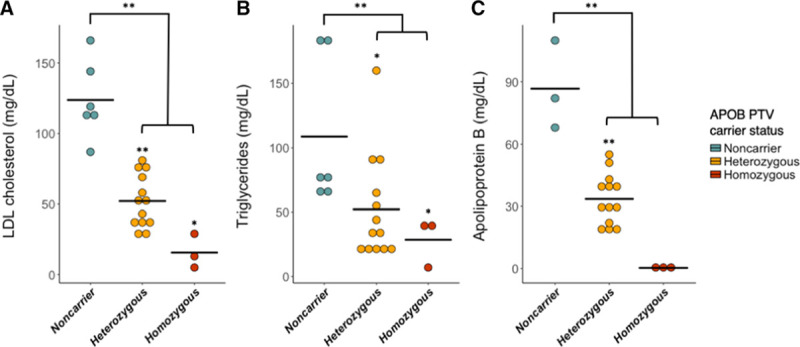
**Serum lipid levels among Japanese individuals**. LDL-C (low-density lipoprotein cholesterol; (**A**), triglyceride (**B**), and apoB (**C**) are compared among heterozygous (n=13) and homozygous (n=3) *APOB* protein truncating variant carriers and noncarriers (n=6). Each dot represents an individual’s lipid level. Each horizontal line indicates mean value of the lipid level for each genotype. *P* values were calculated using Mann-Whitney *U* test. PTV indicates protein truncating variant. *P<0.05, **P<0.01 compared with noncarriers.

In FHBL pedigrees, we tested whether *APOB* PTVs were associated with serum lipids and apolipoproteins. We recruited 29 Japanese FHBL families and sequenced the exome in 69 participants from the families. Of those, 12 individuals in 4 families and 4 single probands harbored *APOB* PTVs that appeared causative (Figure I in the Data Supplement). Among these individuals, 3 carried PTVs in homozygous state and 13 harbored PTVs in heterozygous form. Identified causative variants were confirmed through Sanger sequencing (primers shown in Table I in the Data Supplement). Five of these *APOB* PTVs had not been previously described in FHBL families (Table II in the Data Supplement). The *APOB* PTVs cosegregated with serum LDL-C and apoB levels. Both homozygote and heterozygous carriers exhibited reduction of serum LDL-C, triglyceride, and apoB levels (Figure 1, Table III in the Data Supplement). Based on linear regression for effect size (95% CI), carrying a PTV in *APOB* was associated with lower LDL-C (−55 mg/dL; 95% CI, −68 to −42; Mann-Whitney *U P*=2.7×10^-5^), lower triglyceride levels (−53%; 95% CI, −72 to −21; Mann-Whitney *U P*=1.7×10^-4^), and lower apoB (−43 mg/dL; 95% CI, −53 to −33; Mann-Whitney *U P*=2.1×10^-3^) after adjusting for age and sex.

In the set of Japanese FHBL individuals, *APOB* PTV carriers had higher hepatobiliary enzymes compared with noncarriers (Table III in the Data Supplement). The 3 individuals homozygous for *APOB* PTV were all >40 years old with evidence of fatty liver on imaging and associated elevation in hepatobiliary enzymes (Table IV in the Data Supplement).

### Association of *APOB* PTVs With Lipids and CHD

We sequenced the *APOB* gene in a total of 57 973 participants from the MIGen (Myocardial Infarction Genetics Consortium) of African, European, and South Asian ancestries (N=33 835) and from participants of European ancestry (N=24 138) in the Geisinger Health System and Regeneron Genetics Center DiscovEHR study who were recruited as part of the MyCode Community Health Initiative^9^ (Table 1). Across a total of 57 973 individuals in 12 studies (Table V in the Data Supplement), we observed 37 *APOB* PTVs. Thirty-two (86%) of these PTVs were only seen in a single individual (Table VI in the Data Supplement). These mutations included 19 nonsense single-nucleotide substitutions, 3 single-nucleotide substitutions that were predicted to disrupt splicing, and 15 frameshift indels. In aggregate, these 37 mutations were seen in a total of 56 individuals in heterozygous form. No homozygotes or compound heterozygotes were observed.

**Table 1.**
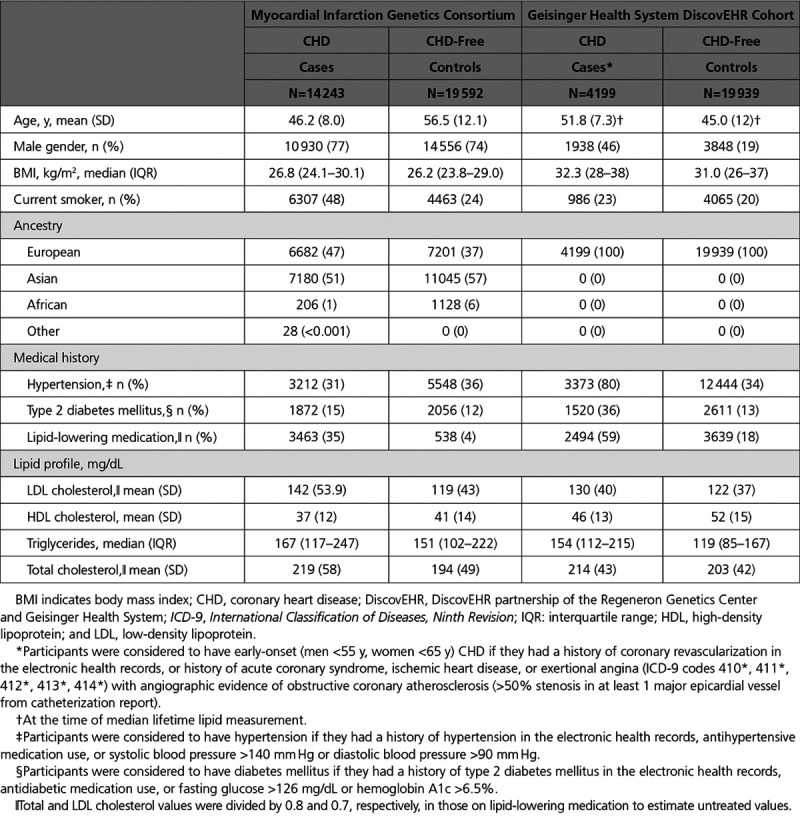
Baseline Characteristics of Myocardial Infarction Genetics Consortium and DiscovEHR Study Participants

Among MIGen individuals free of CHD, we found that *APOB* PTV carriers had 43 mg/dL lower LDL-C (95% CI, −59.4 to −26.9; *P*=2.1×10^-7^), 53 mg/dL lower total cholesterol (95% CI, −72.4 to −34.3; *P*=4.2×10^-8^), 4 mg/dL higher HDL-C (high-density lipoprotein cholesterol; 95% CI, −0.39 to 8.8; *P*=0.07), and 32% lower triglycerides (95% CI, 15%–45%; *P*=5.0×10^-4^; Table 2). Additionally, among 37 912 individuals in DiscovEHR, *APOB* PTV carriers had a 48 mg/dL lower LDL-C (95% CI, −61.9 to −33.4; *P*=5.6×10^-11^).

**Table 2.**
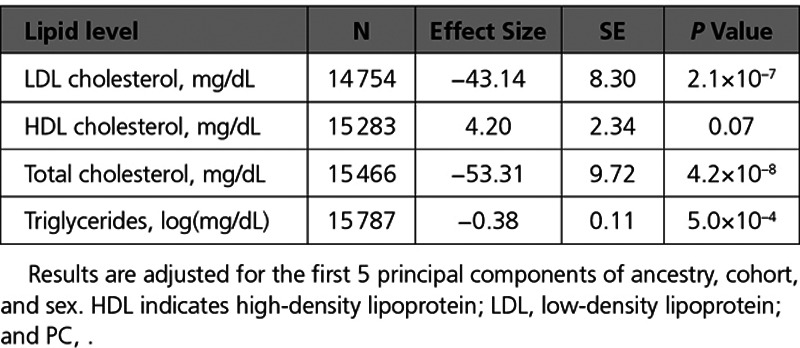
Associations of *APOB* Protein Truncating Variant Carrier Status With Plasma Lipids in the Myocardial Infarction Genetics Consortium

**Figure 2.**
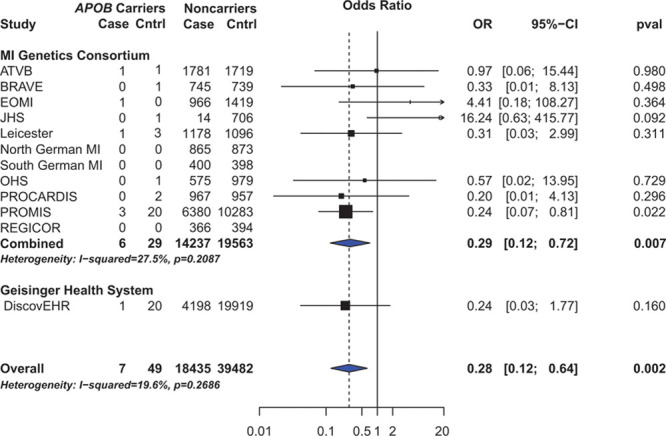
**Association of *APOB* protein truncating variant carrier status with risk of coronary heart disease (CHD) among 57 973 individuals.** In each study, the relationship of protein truncating variants in *APOB* with risk of CHD was determined. Exact methods were used to calculate *P* values for association tests and CI. Cochran-Mantel-Haenszel statistics for stratified 2-by-2 tables was performed for meta-analysis. Odds ratio in the North German MI study (North German Myocardial Infarction) and South German MI study (South Germ Myocardial Infarction) were not available due to a lack of observed *APOB* protein truncating variant carriers. ATVB indicates Italian Atherosclerosis, Thrombosis, and Vascular Biology; BRAVE, Bangladesh Risk of Acute Vascular Events study; Cntrl, control; DiscovEHR, DiscovEHR partnership of the Regeneron Genetics Center and Geisinger Health System; EOMI, Exome Sequencing Project Early-Onset Myocardial Infarction; JHS, Jackson Heart Study; Leicester, Leicester Myocardial Infarction; MI, myocardial infarction; OHS, Ottawa Heart Study; OR, odds ratio; PROCARDIS, Precocious Coronary Artery Disease; PROMIS, Pakistan Risk of Myocardial Infarction Study; REGICOR, Registre Gironí del COR (Gerona Heart Registry).

Among the 18 442 individuals with CHD, 7 individuals carried a PTV in *APOB* (0.038% carrier frequency) compared with 49 of the 39 531 controls (0.092% carrier frequency; Figure 2). Carriers of *APOB* PTVs had 72% lower risk of CHD when compared with noncarriers (odds ratio, 0.28; 95% CI, 0.12–0.64; *P*=0.002). In a sensitivity analysis, we found similar results (odds ratio, 0.29; 95% CI, 0.12–0.71; *P*=0.006) in the MIGen study after adjusting for sex, principal components (PCs) of ancestry, and cohort.

## Discussion

In this study, we assessed whether rare PTVs in *APOB* were associated with lower lipid levels and reduced CHD. Among Japanese FHBL families, we found that carrying an *APOB* PTV in heterozygous form was associated with lower apoB, LDL-C, and triglycerides. Among >57 000 participants with and without CHD, *APOB* PTV carrier status also linked to lower total cholesterol, LDL-C, triglycerides, and a 72% lower risk for CHD when compared with noncarriers. These results permit several conclusions.

First, we demonstrate that *APOB* PTVs are a frequent cause of FHBL among the Japanese in this study. By analyzing 29 pedigrees with an extreme LDL-C phenotype, we identified 13 heterozygous carriers and 3 homozygous carriers. Identification of such individuals can enable deep phenotyping to understand the consequences of lifelong perturbation. For example, we note that each of the 3 homozygotes had not only extremely low LDL-C but also evidence of fatty liver. The presence of fatty liver is consistent with previous reports of adverse effects of using *APOB* inhibitors.^10,11^

Second, we provide evidence that, despite an increased risk of fatty liver, carriers of *APOB* PTVs are at substantially reduced risk of CHD. These findings are of particular importance because clinical trials of mipomersen for CHD outcomes are highly unlikely to be undertaken because of the associated adverse liver effects of mipomersen. These results emphasize the dominant role of apoB-containing lipoproteins in protection from CHD.

Third, our results add to a growing body of evidence demonstrating that rare variants associated with reduced circulating apoB-containing lipoproteins are associated with reduced risk of CHD. Rare nonsense mutations in the *PCSK9* (proprotein convertase subtilisin/kexin type 9) gene was noted in 2.6% of blacks and associated with a 88% reduction in risk for CHD.^12^ Also, *NPC1L1* (NPC1 like intracellular cholesterol transporter 1) rare inactivating variants are observed 1 in 650 individuals and linked to a 53% relative risk reduction for CHD.^13^

Strengths of this study include the large sample size and the evaluation of family-based and population-based samples. However, we were not able to assess hepatic enzymes in the population-based samples, we did not functionally validate PTVs, and we were unable to compare effects stratified by ancestry groups given the small number of individuals carrying PTVs within each study.

## Conclusions

Rare PTVs in the *APOB* gene associated with lower LDL-C, lower triglycerides, and decreased risk for CHD.

## Sources of Funding

Dr Peloso is supported by the National Heart, Lung, and Blood Institute (NHLBI) of the National Institutes of Health under Award Number K01HL125751. Dr Nomura was supported by the Yoshida Scholarship Foundation. Dr Khera is supported by an institutional grant from the Broad Institute of Massachusetts Institute of Technology (MIT) and Harvard (BroadIgnite), a K08 from the National Human Genome Research Institute (K08HG010155), and a Junior Faculty Award from the National Lipid Association. Dr Kathiresan is supported by a research scholar award from the Massachusetts General Hospital, the Donovan Family Foundation, and grant R01 HL127564 from the NHLBI. Funding for the EOMI study (Exome Sequencing Project Early-Onset Myocardial Infarction) was provided by grants RC2 HL103010 (HeartGO, Heart Grand Opportunity), RC2 HL102923 (LungGO, Lung Grand Opportunity), and RC2 HL102924 (WHISP) from the NHLBI. Exome sequencing was performed through grants RC2 HL102925 (BroadGO, Broad Grand Opportunity) and RC2 HL102926 (SeattleGO, Seattle Grand Opportunity) from the NHLBI. Exome sequencing in ATVB (Italian Atherosclerosis, Thrombosis, and Vascular Biology), the PROCARDIS study (Precocious Coronary Artery Disease), the OHS (Ottawa Heart Study), PROMIS (Pakistan Risk of Myocardial Infarction Study), South German MI study (South German Myocardial Infarction), and the JHS (Jackson Heart Study) was supported by grant 5U54HG003067 from the National Institutes of Health. Fieldwork, genotyping, and standard clinical chemistry assays in PROMIS were principally supported by grants awarded to the University of Cambridge from the British Heart Foundation, UK Medical Research Council, Wellcome Trust, European Union (EU) Framework 6–funded Bloodomics Integrated Project, Pfizer, Novartis, and Merck. Additional support for PROMIS was provided by the UK Medical Research Council (MR/L003120/1), British Heart Foundation (RG/13/13/30194), UK National Institute for Health Research Cambridge Biomedical Research Centre, European Research Council (268834), and European Commission Framework Programme 7 (HEALTH-F2-2012–279233). The Jackson Heart Study is supported by contracts HHSN268201300046C, HHSN268201300047C, HHSN268201300048C, HHSN268201300049C, and HHSN268201300050C from the NHLBI and the National Institute on Minority Health and Health Disparities. Dr Wilson is supported by U54GM115428 from the National Institute of General Medical Sciences. REGICOR study (Registre Gironí del COR [Gerona Heart Registry]) was supported by the Spanish Ministry of Economy and Innovation through the Carlos III Health Institute (Red Investigación Cardiovascular RD12/0042, PI09/90506), European Funds for Development (ERDF-FEDER), and by the Catalan Research and Technology Innovation Interdepartmental Commission (2014SGR240). Samples for the Leicester (Leicester Myocardial Infarction) cohort were collected as part of projects funded by the British Heart Foundation (British Heart Foundation Family Heart Study, RG2000010; UK Aneurysm Growth Study, CS/14/2/30841) and the National Institute for Health Research (NIHR Leicester Cardiovascular Biomedical Research Unit Biomedical Research Informatics Centre for Cardiovascular Science, IS_BRU_0211_20033). The South MI Study is supported by the German Federal Ministry of Education and Research (BMBF) in the context of the e:Med program (e:AtheroSysMed) and the FP7 European Union project CVgenes@target (261123). Additional grants were received from the Fondation Leducq (CADgenomics: Understanding Coronary Artery Disease Genes, 12CVD02). This study was also supported through the Deutsche Forschungsgemeinschaft cluster of excellence Inflammation at Interfaces and SFB 1123. The ATVB study was supported by a grant from RFPS-2007-3-644382 and Programma di ricerca Regione-Università 2010–2012 Area 1–Strategic Programmes–Regione Emilia-Romagna. The authors would like to thank the MyCode Community Health Initiative participants for their permission to utilize their health and genomics information in the DiscovEHR (DiscovEHR partnership of the Regeneron Genetics Center and Geisinger Health System) collaboration. The DiscovEHR study was funded, in part, by the Regeneron Genetics Center.

## Disclosures

Dr Kathiresan reports grant support from Regeneron and Bayer, grant support and personal fees from Aegerion, personal fees from Regeneron Genetics Center, Merck, Celera, Novartis, Bristol-Myers Squibb, Sanofi, AstraZeneca, Alnylam, Color Genomics, Corvidia, Eli Lilly, and Leerink Partners, personal fees and other support from Catabasis, and other support from San Therapeutics outside the submitted work. He holds equity in Verve Therapeutics and Maze Therapeutics. He is also the chair of the scientific advisory board at Genomics plc. Drs Teslovich, Shane McCarthy, Baras, and Dewey are employees of Regeneron Pharmaceuticals. The views expressed in this article are those of the authors and do not necessarily represent the views of the NHLBI; the National Institutes of Health; or the US Department of Health and Human Services

## Supplementary Material
