# Supplementary material for: Rare Protein-Truncating Variants in APOB, Lower Low-Density Lipoprotein Cholesterol, and Protection Against Coronary Heart Disease
Source: Circ Genom Precis Med. 2019 May 21;12(5):e002376. doi: 10.1161/CIRCGEN.118.002376 (PMC7044908; doi:10.1161/CIRCGEN.118.002376)
Supplement: Supplementary file 1 [file hcg-12-e002376-s001.pdf]

SUPPLEMENTAL MATERIAL

Table of Contents

Methods 2

Supplemental Table 1. Primer sequences used for direct sequencing 7

Supplemental Table 2. *APOB* causative protein-truncating variants from hypobetalipoproteinemia families 8

Supplemental Table 3. Clinical characteristics of hypobetalipoproteinemia families by *APOB* variant carrier status 9

Supplemental Table 4. Clinical characteristics of homozygous *APOB* PTV carriers 10

Supplemental Table 5. Definitions of coronary heart disease (CHD) across studies 11

Supplemental Table 6. *APOB* protein truncating variants in CHD cases and controls 13

Supplemental Figure 1. Pedigrees of Hypobetalipoproteinemia families 14

Supplemental References 15

METHODS

Study participants

We recruited twenty-nine FHBL pedigrees to study associations of *APOB* PTV carrier status with lipid profiles from the Kanazawa University Mendelian Disease Registry in Japan. Participants were diagnosed with FHBL if low serum LDL-C or apoB levels were observed (LDL-C < 70 mg/dL or apoB < 50 mg/dL). We used these thresholds of LDL-C and apoB for FHBL diagnosis in order not to miss detecting putative FHBL individuals harboring *APOB* PTVs [1]. Causative variants were identified by whole exome sequencing and *APOB* PTVs co-segregating with phenotype within each pedigree were identified. Identified causative variants were then confirmed through Sanger sequencing (primers shown in **Supplemental Table 1**).

Additionally, we sequenced the *APOB* gene in a total of 57,973 participants from the Myocardial Infarction Genetics Consortium (MIGen) of African, European, and South Asian ancestries (N=33,835), and from participants of European ancestry (N=24,138) in the Geisinger Health System and Regeneron Genetics Center DiscovEHR study who were recruited as part of the MyCode Community Health Initiative [2] (**Table 1**). MIGen studies included the Italian Atherosclerosis Thrombosis and Vascular Biology (ATVB) study [3], Bangladesh Risk of Acute Vascular Events study (BRAVE) [4], the Exome Sequencing Project Early-Onset Myocardial Infarction (ESP-EOMI) study [5], a nested case-control cohort of the Jackson Heart Study (JHS) [6], the South German Myocardial Infarction study [7], the Ottawa Heart Study (OHS) [8], the Precocious Coronary Artery Disease Study (PROCARDIS) [9], the Pakistan Risk of Myocardial Infarction Study (PROMIS) [10], the Registre Gironi del COR (Gerona Heart Registry or REGICOR) study [11], the Leicester Myocardial Infarction study [12], and the North German

Myocardial Infarction study [13] (**Supplemental Table 5**). Clinical data were assessed in each study.

All participants in the study provided written informed consent for genetic studies. The institutional review boards at the Broad Institute and each participating institution approved the study protocol.

In order to minimize the possibility of unintentionally sharing information that can be used to re-identify private information, a subset of the data generated for this study are available at dbGaP and can be accessed at through dbGaP Study Accessions: phs000814.v1.p1 (ATVB), phs001398.v1.p1 (BRAVE), phs000279.v2.p1 (EOMI), phs001098.v1.p1 (JHS), phs001000.v1.p1 (Leicester), phs000990.v1.p1 (NorthGermanMI), phs000916.v1.p1 (SouthGermanMI), phs000806.v1.p1 (OHS), phs000883.v1.p1 (PROCARDIS), phs000917.v1.p1 (PROMIS), phs000902.v1.p1(Regicor).

Phenotypes

In FHBL pedigrees, all blood samples were obtained after a 12-hour overnight fast. Apolipoprotein B was analyzed by a commercial immunoturbidimetric assay (Apo B auto N Daiichi, Sekisui Medical, Tokyo, Japan)[14]. Fatty liver was diagnosed by an independent liver ultrasound specialist when a participant was observed to have both liver brightness and hepato-renal echo contrast.

In MIGen, fasting LDL-C in mg/dL was used from the earliest available exam in each contributing study. LDL-C was calculated using the Friedewald equation [15, 16] (LDL-C = total cholesterol – high-density lipoprotein cholesterol [HDL-C] – (triglycerides/5)) for those with triglycerides <400 mg/dL. If triglycerides ≥400 mg/dL, calculated LDL-C was set to missing. In the DiscovEHR study, median lipid levels were calculated for each individual following removal

of values that were > 3 standard deviations from the intra-individual median value for individuals with two or more measurements in the EHR [2]. In both MIGen and DiscovEHR, for those on lipid-lowering drug treatment, we replaced LDL-C by a value of the measured LDL-C divided by 0.7 and total cholesterol by a value of the total cholesterol divided by 0.8[17, 18]. HDL-C and triglyceride levels were not adjusted for lipid-altering medication use, and triglyceride levels were natural logarithm transformed for statistical analysis.

In MIGen, early-onset CHD was defined as myocardial infarction, angiographic coronary artery disease, coronary artery bypass surgery, or percutaneous coronary revascularization in men ≤50 years or women ≤60 years. Details for clinical phenotypes for each cohort are available in **Supplemental Table 5**. In DiscovEHR, the present analysis was restricted to early-onset CHD cases and CHD-free controls (age <55 years for men or <65 years for women for both cases and controls). Participants were considered to have CHD if they had a history of coronary revascularization in the EHR, or history of acute coronary syndrome, ischemic heart disease, or exertional angina (ICD-9 codes 410*, 411*, 412*, 413*, 414*) with angiographic evidence of obstructive coronary atherosclerosis (>50% stenosis in at least one major epicardial vessel from catheterization report). CHD-free controls were defined as individuals without any case criteria or any single encounter or problem list diagnosis code indicating CHD.

Gene sequencing

Whole exome sequencing of MIGen was performed at the Broad Institute (Cambridge, MA, USA) as previously described [5]. Sequencing reads were aligned to a human reference genome (build 37) using the Burrows–Wheeler Aligner-Maximal Exact Match algorithm. Aligned non-duplicate reads were locally realigned, and base qualities were recalibrated using the Genome Analysis ToolKit (GATK) software [19]. Variants were jointly called using the GATK

HaplotypeCaller program. The sensitivity of variant quality score recalibration (VQSR) threshold was 99.6% for single nucleotide variants and 95% for insertion/deletion variants, as we have previously reported[20]. We annotated all identified variants with the use of the Variant Effect Predictor software (version 88) [21, 22]. PTV were defined as high confidence (https://github.com/konradjk/loftee) nonsense, splice-site, and frameshift mutations with minor allele frequency < 1% across contributing cohorts.

In the DiscovEHR study, *APOB* sequences were extracted from whole exome sequences generated as previously described [2]. Sequence reads were aligned to the human reference build GRCh37.p13. Single nucleotide variants (SNV) and insertion/deletion (indel) sequence variants were identified using the Genome Analysis Toolkit [23] and annotated using SnpEff[24]. PTVs were defined as any of the following: SNVs leading to a premature stop codon, loss of a start codon, or loss of a stop codon; SNVs or indels disrupting canonical splice acceptor or donor dinucleotides; open reading frame shifting indels leading to the formation of a premature stop codon.

Statistical Analysis

In FHBL pedigrees, the differences in cholesterol and hepatobiliary enzymes stratified by *APOB* PTV carrier status were analyzed using the *Mann-Whitney U* test while effect sizes were obtained from a linear regression associating carrying an *APOB* PTV on cholesterol adjusted for age and sex.

We performed linear regression with controls to associate *APOB* PTV carrier status with each blood lipid level in the MIGen studies and with LDL-C in the DiscovEHR study adjusting for age, sex, the first 5 principle components of ancestry, and indicators of cohort status.

In order to associate an aggregate of PTVs in the *APOB* gene with CHD risk, we performed an exact Cochran-Mantel-Haenszel analysis for stratified 2-by-2 tables [25] implemented in the meta R package. Heterogeneity was measured by the I^2 statistic, calculated in the meta R package. It describes the percentage of variation in association statistics across studies that is due to heterogeneity of the statistics rather than due to chance. We obtained p-values for proportion of null allele counts in cases versus controls and odds ratios (OR) with 95% confidence intervals (CI). We considered a p-value less than 0.05 as statistically significant. Additionally, since the Cochran-Mantel-Haenszel test does not allow adjustment for covariates, we performed a sensitivity analysis in the MIGen study using logistic regression adjusting for cohort, sex, and the first 4 principal components (PCs) of ancestry to control for potential population stratification[26, 27]. The first 4 PCs of ancestry had p-values < 0.01 for association with CHD after adjusting for the cohort and sex.

The R software (The R Project for Statistical Computing, Vienna, Austria) was used for all analyses.

Supplemental Table 1. Primer sequences used for direct sequencing.

CHR:BP (b37)	REF	ALT	Primer (Forward)	Primer (Reverse)
2:21228306	C	CA	TCCAAAGCAGCAATGCCATC	TGCCCTCAACCTACCAACAC
2:21228457	G	T	TTTGGAAGCGTGAACTGGGA	ACACCAAAAACCCCAATGGC
2:21233797	C	T	CCAGTAAGCTCCACGCCAAT	ACAAAGGCTCCACAAGTCATCA
2:21235299	CAA	C	TTGGACTCTCCATTGAGCCG	GTTCCTGGGGACCACAGATG
2:21242647	TG	T	GTCAGCGGATAGTAGGAGGC	GGTCAGTTTGCAAGCAAGTC
2:21250863	CGA	C	GGCTGGGTCAAGTGATGGAA	TCCAACTGTCATGGACTTCAGA
2:21251197	A	C	CAGGGCCCTCAGTGGTATATG	TCCTCTTTTGACTGCAGGACC
2:21260973	T	A	CCGGGTAAAGGAAAACCTGCT	ACCATCCTCTCTCTGGGACA

Abbreviations: ALT, alternative allele; BP (b37), base position build GRCh37; CHR, chromosome; and REF, reference allele.

Supplemental Table 2. ***APOB*** **causative protein-truncating variants from hypobetalipoproteinemia families.**

CHR:BP (b37)	REF	ALT	rsID	AA change	Consequence	gnomAD EAS MAF	FHBL Family #	Comment
2:21228306	C	CA	.	E3812fs	Frameshift	NA	10	Reported
2:21228457	G	T	rs757204163	C3761X	Premature stop	0	9	Reported
2:21233797	C	T	.	W1981X	Premature stop	NA	28	Novel
2:21235299	CAA	C	.	F1480fs	Frameshift	NA	12	Novel
2:21242647	TG	T	.	N983fs	Frameshift	NA	4	Novel
2:21250863	CGA	C	.	R635fs	Frameshift	NA	23	Reported
2:21251197	A	C	.	c.1829+2T>G	splice donor	NA	29	Novel
2:21260973	T	A	.	K132X	Premature stop	NA	6	Novel

Abbreviations: AA, amino acid; ALT, alternative allele; BP (b37), base position build GRCh37; CHR, chromosome; EAS, East Asians; FHBL, familial hypobetalipoproteinemia; gnomAD, the Genome Aggregation Database; MAF, minor allele frequency; and REF, reference allele.

Supplemental Table 3. Clinical characteristics of hypobetalipoproteinemia families by *APOB* variant carrier status.

	APOB PTV carrier					
	**Non-carrier**	**Heterozygous**	**Homozygous**	**Non-carrier vs. Hetero p-value***	**Non-carrier vs. Homo p-value***	**Non-carrier vs. Carriers p-value***
**N**	6	13	3			
**Age, mean±SD**	52.3 ± 29	46.4 ± 26	41.7 ± 0.58			
**Male sex, n(%)**	2 (33)	8 (62)	1 (33)			
**Regular alcohol intake, n(%)**	0 (0)	2 (15)	1 (33)			
**Lipids (mg/dL)**						
LDL cholesterol	116 (113–138)	52 (37–69)	13 (9–21)	7.4×10^{-5}	0.023	2.7×10^{-5}
HDL cholesterol	55 (44–60)	63 (56–77)	49 (46–69)	0.25	0.90	0.30
Triglyceride	77 (70–157)	36 (21–65)	37 (22–40)	0.022	0.024	7.9×10^{-3}
Total cholesterol	197 (187–212)	125 (98–139)	86 (71–95)	4.8×10^{-4}	0.024	1.7×10^{-4}
**Lipoprotein (mg/dL)**						
Apolipoprotein B	82 (75–96)	31 (22–41)	0 (0–0.5)	3.6×10^{-3}	0.10	2.1×10^{-3}
**Hepatobiliary enzymes (U/L)**						
AST	21 (19–30)	44 (38–58)	40 (30–42)	5.9×10^{-3}	0.25	7.5×10^{-3}
ALT	30 (20–34)	55 (41–70)	39 (28–53)	6.4×10^{-3}	0.64	0.018
gamma GTP	31 (30–40)	61 (37–67)	70 (44–76)	0.14	0.57	0.15

Continuous variables are presented as median (IQR) unless otherwise noted.
Dichotomous variables are presented as n (%).
ALT, alanine aminotransferase; AST, aspartate aminotransferase; GTP, glutamyl transpeptidase; IQR, interquartile range.
*: P-values were calculated using *Mann-Whitney U test*.

Supplemental Table 4. Clinical characteristics of homozygous *APOB* PTV carriers.

	Ind #1	Ind #2	Ind# 3
APOB variant	p.C3761X	p.E3812fs	p.Q1981X
Sex	Female	Male	Female
Age, years	41	42	42
Regular alcohol intake	No	Yes	No
Fatty Liver	Yes	Yes	Yes
Eye problem	No	No	No
Neurological dysfunction	No	No	No
Hepatobiliary enzymes (U/L)			
AST	20	44	40
ALT	17	66	39
Gamma GTP	17	82	70

ALT, alanine aminotransferase; AST, aspartate aminotransferase; GTP, glutamyl transpeptidase; IQR, interquartile range.

Supplemental Table 5. Definitions of coronary heart disease (CHD) across studies.

Study	Ancestry	Country of Origin	CHD Cases	Controls	CHD Definition	Control Definition
ATVB	European	Italy	1782	1720	MI in male or female ≤ 45y	No history of thromboembolic disease
BRAVE	South Asian	Bangladesh	745	740	MI in men and women ≤60y	Controls without CAD; men and women ≤65y
DiscovEHR	European	USA	4199	19939	History of coronary revascularization, acute coronary syndrome, ischemic heart disease, or exertional angina with angiographic evidence of obstructive coronary disease (>50% stenosis in at least one major epicardial vessel) in men <55y or women <65y	Absence of CAD case criteria or electronic health record problem list diagnosis code indicating CAD
ESP-EOMI	European African-American	USA	967	1419	EOMI (male ≤ 50y or female ≤ 60y)	Hospital-based, no report of MI by history
JHS	African-American	USA	14	707	Combination of prevalent CHD (self-reported or electrocardiographic evidence of MI) and incident CHD (MI or coronary revascularization) in male ≤ 50y or female ≤ 60y.	Free of CHD during > 14 y follow-up
Leicester	European African South Asian	UK	1179	1099	MI in men or women age ≤60y	Controls ≥64y without reported CAD history
North German MI	European	Germany	865	873	MI in men and women ≤60y	Controls without CAD; men and women ≤65y
South German MI	European	Germany	400	398	MI in men ≤40y or women ≤55y	Controls without CAD, men ≥ 65y and women ≥75y
OHS	European	Canada	575	980	MI or CABG or angiographic disease (>50% stenosis) in men ≤50y or women ≤60y), without type 2 diabetes	Asymptomatic
PROCARDIS	European	Multiple European	967	959	MI (men ≤50y or women ≤60y)	No history of CAD
PROMIS	South Asian	Pakistan	6383	10303	MI, age ≤50y	Age and gender frequency-matched. No history of MI/CVD
REGICOR	European	Spain	366	394	MI, male ≤ 50y or female ≤ 60y)	Controls from a population- based study; free of MI, coronary revascularization; ≥ 55 and <80y

Abbreviations: CAD, coronary artery disease; CHD, coronary heart disease; CABG, Coronary artery bypass grafting; CVD, cardiovascular disease; EOCAD, early-onset coronary artery disease; EOMI, early-onset myocardial infarction; MI, myocardial infarction.

Supplemental Table 6. *APOB* protein truncating variants in CHD cases and controls.

POS (hg19)	REF	ALT	Type	Amino Acid Change	*MIGen CHD (n=14,243)*	*MIGen Controls (n=19,592)*	*DiscovEHR CHD (n=4,199)*	*DiscovEHR Controls (n=19,939)*
2:21225555	G	A	stop gained	p.Gln4247Ter	1	0	0	0
2:21225715	G	GA	frameshift	p.Ile4194HisfsTer2	3	7	0	0
2:21225598	A	T	stop gained	p.Tyr4232Ter	0	0	0	1
2:21225753	C	A	stop gained	p.Glu4181Ter	0	3	0	0
2:21225765	G	A	stop gained	p.Arg4177Ter	2	0	0	0
2:21225930	G	A	stop gained	p.Gln4122Ter	0	1	0	0
2:21226023	C	A	stop gained	p.Glu4091Ter	0	1	0	0
2:21226188	G	GT	frameshift		0	0	0	7
2:21227163	C	T	stop gained	p.Trp4022Ter	0	1	0	0
2:21228002	AG	A	frameshift	p.Leu3913TrpfsTer12	0	1	0	0
2:21228027	TG	T	frameshift		0	0	0	2
2:21228410	G	T	stop gained	p.Ser3777Ter	0	1	0	0
2:21228615	CA	C	frameshift		0	0	0	1
2:21229052	C	T	stop gained	p.Trp3563Ter	0	1	0	0
2:21229480	AT	A	frameshift		0	0	0	1
2:21230482	ACTTG	A	frameshift	p.Ala3085ValfsTer4	0	1	0	0
2:21230620	TGAAAA	T	frameshift	p.Phe3039SerfsTer5	0	0	0	1
2:21230841	G	GT	frameshift		0	0	0	1
2:21230999	AAC	A	frameshift		0	0	0	1
2:21232176	G	A	stop gained	p.Arg2522Ter	0	0	1	0
2:21232182	G	A	stop gained	p.Arg2520Ter	0	1	0	0
2:21232326	C	A	stop gained	p.Glu2472Ter	0	1	0	0
2:21232834	TC	T	frameshift	p.Gly2302GlufsTer9	0	1	0	0
2:21233487	G	A	stop gained	p.Arg2085Ter	0	1	0	0
2:21233596	CT	C	frameshift		0	0	0	1
2:21233706	G	A	stop gained	p.Arg2012Ter	0	1	0	0
2:21233814	C	A	stop gained	p.Glu1976Ter	0	1	0	0
2:21234181	TGCTTTATA	T	frameshift	p.Tyr1851ArgfsTer5	0	0	0	1
2:21234276	GC	G	frameshift	p.His1822MetfsTer6	0	1	0	0
2:21235328	A	T	stop gained	p.Leu1471Ter	0	1	0	0
2:21236107	CTG	C	frameshift		0	0	0	1
2:21241899	A	T	stop gained	p.Leu1029Ter	0	1	0	0
2:21246396	C	A	splice donor		0	0	0	1
2:21246396	C	T	splice donor		0	0	0	1
2:21250698	A	C	splice donor		0	1	0	0
2:21255263	G	A	stop gained	p.Arg439Ter	0	1	0	0
2:21257742	G	A	stop gained	p.Gln284Ter	0	1	0	0

Supplemental Figure 1. Pedigrees of Hypobetalipoproteinemia families.
Square and circle indicate male and female. Black half indicates heterozygote subjects; black, homozygote subjects; gray shading, genetically unknown subjects; and white, genetically unaffected subjects. Total cholesterol (mg/dL), triglycerides (mg/dL), high-density lipoprotein cholesterol (mg/dL), and low-density lipoprotein cholesterol (mg/dL) levels are displayed below each individual identifier.

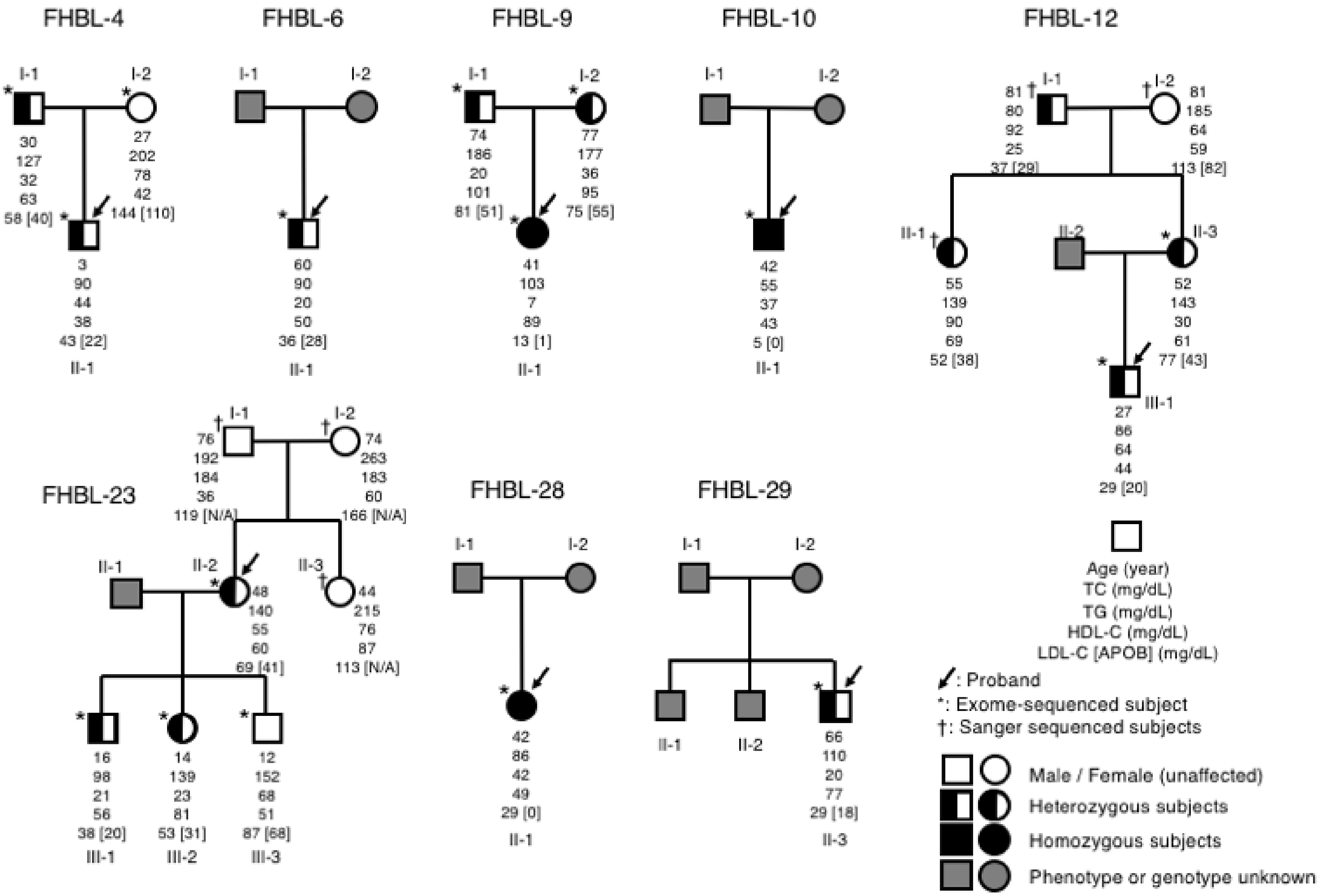

Supplemental References

1. Katsuda S, et al. Apolipoprotein B gene mutations and fatty liver in Japanese hypobetalipoproteinemia. *Clinica chimica acta; international journal of clinical chemistry*. 2009;399:64-8.
2. Dewey FE, et al. Inactivating Variants in ANGPTL4 and Risk of Coronary Artery Disease. *N Engl J Med*. 2016;374:1123-33.
3. Atherosclerosis, Thrombosis, and Vascular Biology Italian Study Group. No evidence of association between prothrombotic gene polymorphisms and the development of acute myocardial infarction at a young age. *Circulation*. 2003;107:1117-22.
4. Chowdhury R, et al. The Bangladesh Risk of Acute Vascular Events (BRAVE) Study: objectives and design. *European journal of epidemiology*. 2015;30:577-87.
5. Do R, et al. Exome sequencing identifies rare LDLR and APOA5 alleles conferring risk for myocardial infarction. *Nature*. 2015;518:102-6.
6. Taylor HA, Jr., et al. Toward resolution of cardiovascular health disparities in African Americans: design and methods of the Jackson Heart Study. *Ethn Dis*. 2005;15:S6-4-17.
7. TG and HDL Working Group of the Exome Sequencing Project, National Heart, Lung, and Blood Institute, et al. Loss-of-function mutations in APOC3, triglycerides, and coronary disease. *The New England journal of medicine*. 2014;371:22-31.
8. McPherson R, et al. A common allele on chromosome 9 associated with coronary heart disease. *Science*. 2007;316:1488-91.
9. Clarke R, et al. Genetic variants associated with Lp(a) lipoprotein level and coronary disease. *N Engl J Med*. 2009;361:2518-28.
10. Saleheen D, et al. The Pakistan Risk of Myocardial Infarction Study: a resource for the study of genetic, lifestyle and other determinants of myocardial infarction in South Asia. *Eur J Epidemiol*. 2009;24:329-38.
11. Senti M, et al. Paraoxonase1-192 polymorphism modulates the nonfatal myocardial infarction risk associated with decreased HDLs. *Arterioscler Thromb Vasc Biol*. 2001;21:415-20.
12. Samani NJ, et al. Genomewide association analysis of coronary artery disease. *The New England journal of medicine*. 2007;357:443-53.
13. Myocardial Infarction Genetics and CARDIoGRAM Exome Consortia Investigators, et al. Coding Variation in ANGPTL4, LPL, and SVEP1 and the Risk of Coronary Disease. *The New England journal of medicine*. 2016;374:1134-44.
14. Sakurabayashi I, et al. Reference intervals for serum apolipoproteins A-I, A-II, B, C-II, C-III, and E in healthy Japanese determined with a commercial immunoturbidimetric assay and effects of sex, age, smoking, drinking, and Lp(a) level. *Clinica chimica acta; international journal of clinical chemistry*. 2001;312:87-95.
15. Friedewald WT, et al. Estimation of the concentration of low-density lipoprotein cholesterol in plasma, without use of the preparative ultracentrifuge. *Clinical chemistry*. 1972;18:499-502.
16. Warnick GR, et al. Estimating low-density lipoprotein cholesterol by the Friedewald equation is adequate for classifying patients on the basis of nationally recommended cutpoints. *Clinical chemistry*. 1990;36:15-9.
17. Tobin MD, et al. Adjusting for treatment effects in studies of quantitative traits: antihypertensive therapy and systolic blood pressure. *Statistics in medicine*. 2005;24:2911-35.
18. Peloso GM, et al. Association of Low-Frequency and Rare Coding-Sequence Variants with Blood Lipids and Coronary Heart Disease in 56,000 Whites and Blacks. *American journal of human genetics*. 2014;94:223-232.
19. DePristo MA, et al. A framework for variation discovery and genotyping using next-generation DNA sequencing data. *Nature genetics*. 2011;43:491-8.
20. Khera AV, et al. Association of Rare and Common Variation in the Lipoprotein Lipase Gene With Coronary Artery Disease. *JAMA*. 2017;317:937-946.
21. McLaren W, et al. Deriving the consequences of genomic variants with the Ensembl API and SNP Effect Predictor. *Bioinformatics*. 2010;26:2069-70.

22. McLaren W, et al. The Ensembl Variant Effect Predictor. *Genome Biol*. 2016;17:122.
23. McKenna A, et al. The Genome Analysis Toolkit: a MapReduce framework for analyzing next-generation DNA sequencing data. *Genome Res*. 2010;20:1297-303.
24. Cingolani P, et al. A program for annotating and predicting the effects of single nucleotide polymorphisms, SnpEff: SNPs in the genome of Drosophila melanogaster strain w1118; iso-2; iso-3. *Fly (Austin)*. 2012;6:80-92.
25. Mantel N and Haenszel W. Statistical aspects of the analysis of data from retrospective studies of disease. *Journal of the National Cancer Institute*. 1959;22:719-48.
26. Patterson N, et al. Population structure and eigenanalysis. *PLoS genetics*. 2006;2:e190.
27. Price AL, et al. Principal components analysis corrects for stratification in genome-wide association studies. *Nature genetics*. 2006;38:904-9.
