# Supplementary material for: Rare Protein-Truncating Variants in APOB, Lower Low-Density Lipoprotein Cholesterol, and Protection Against Coronary Heart Disease
Source: Circ Genom Precis Med. 2019 May 21;12(5):e002376. doi: 10.1161/CIRCGEN.118.002376 (PMC7044908; doi:10.1161/CIRCGEN.118.002376)
Supplement: Supplementary file 2 [file hcg-12-e002376-s002.pdf]

Rare Protein-truncating Variants in *APOB*, Lower LDL-C, and Protection Against Coronary Heart Disease

Running title: *Peloso & Nomura et al.; APOB variants with LDL and CHD*

Gina M. Peloso, PhD[1]*, Akihiro Nomura, MD, PhD[2]*, Amit V. Khera, MD[3-6], Mark Chaffin, MS[3], Hong-Hee Won, PhD[7], Diego Ardissino, MD[8,9], John Danesh, FMedSci[10-12], Heribert Schunkert, MD[13], James G. Wilson, MD[14], Nilesh Samani, FMedSci[15,16], Jeanette Erdmann, PhD[17], Ruth McPherson, MD[18], Hugh Watkins, MD, PhD[19], Danish Saleheen, MD[20], Shane McCarthy, PhD[21], Tanya M Teslovich, PhD[21], Joseph B Leader, BA[22], H. Lester Kirchner, PhD[22], Jaume Marrugat, MD, PhD[23,24], Atsushi Nohara, MD, PhD[2], Masaaki Kawashiri, MD[2], Hayato Tada, MD, PhD[2], Frederick E. Dewey, MD[21], David J Carey, PhD[22], Aris Baras, MD[21], Sekar Kathiresan, MD[3-6]

[1]Dept of Biostatistics, Boston Univ School of Public Health, Boston, MA; [2]Dept of Cardiovascular & Internal Medicine, Kanazawa Univ Graduate School of Medicine, Kanazawa, Japan; [3]Program in Medical & Population Genetics, Broad Inst, Cambridge; [4]Cardiovascular Research Ctr, [5]Ctr for Genomic Medicine, [6]Dept of Medicine, Massachusetts General Hospital, Boston, MA; [7]Samsung Advanced Inst for Health Sciences & Technology, Sungkyunkwan Univ, Samsung Medical Ctr, Seoul, Republic of Korea; [8]Cardiology, Azienda Ospedaliero-Universitaria di Parma, Univ of Parma, Parma; [9]ASTC: Associazione per lo Studio Della Trombosi in Cardiologia, Pavia, Italy; [10]MRC/BHF Cardiovascular Epidemiology Unit, Dept of Public Health & Primary Care, [11]The National Inst for Health Research Blood & Transplant Research Unit (NIHR BTRU) in Donor Health & Genomics, Univ of Cambridge, Cambridge; [12]Wellcome Trust Sanger Institute, Genome Campus, Hinxton, UK; [13]Deutsches Herzzentrum München, Technische Universität München, Deutsches Zentrum für Herz-Kreislauf-Forschung, München, Germany; [14]Dept of Physiology & Biophysics, Univ of Mississippi Medical Ctr, Jackson, MS; [15]Dept of Cardiovascular Sciences, Univ of Leicester; [16]NIHR Leicester Biomedical Research Ctr, Glenfield Hospital, Leicester UK; [17]Institute for Integrative & Experimental Genomics, Univ of Lübeck, Lübeck, Germany; [18]Univ of Ottawa Heart Institute, Ottawa, Canada; [19]Cardiovascular Medicine, Radcliffe Dept of Medicine & Wellcome Trust Ctr for Human Genetics, Univ of Oxford, Oxford, UK; [20]Dept of Biostatistics & Epidemiology, Perelman School of Medicine, Univ of Pennsylvania, Philadelphia, PA; [21]Regeneron Genetics Ctr, Tarrytown, NY; [22]Geisinger Health System, Danville, PA; [23]Registre Gironí del Cor group, IMIM (Hospital del Mar Research Institute); [24]CIBER Enfermedades Cardiovasculares (CIBERCV), Barcelona, Spain
*contributed equally

Correspondence:

Sekar Kathiresan

Center for Genomic Medicine

Massachusetts General Hospital

185 Cambridge Street, CPZN 5.830

Boston, MA 02114

Tel: 617-643-6120

Email: skathiresan1@mgh.harvard.edu

Journal Subject Terms: Genetic, Association Studies; Lipids and Cholesterol; Coronary Artery Disease

Abstract:

Background: Familial hypobetalipoproteinemia (FHBL) is a genetic disorder caused by rare protein-truncating variants (PTV) in the gene encoding apolipoprotein B (*APOB*), the major protein component of low-density and triglyceride-rich lipoprotein particles. Whether heterozygous *APOB* deficiency is associated with decreased risk for coronary heart disease (CHD) is uncertain. We combined family-based and large scale gene-sequencing to characterize the association of rare PTVs in *APOB* with circulating low-density lipoprotein cholesterol (LDL-C), triglycerides, and risk for CHD.

Methods: We sequenced the *APOB* gene in 29 Japanese hypobetalipoproteinemia families as well as 57,973 individuals derived from 12 CHD case-control studies – 18,442 with early-onset CHD and 39,531 controls. We defined PTVs as variants that lead to a premature stop, disrupt canonical splice-sites, or lead to insertions/deletions that shift reading frame. We tested the association of rare *APOB* PTV carrier status with blood lipid levels and CHD.

Results: Among 29 FHBL families, 8 families harbored *APOB* PTVs. Carrying one *APOB* PTV was associated with 55 mg/dL lower LDL-C ($p = 3x10^{-5}$) and 53% lower triglyceride level ($p = 2x10^{-4}$). Among 12 case-control studies, an *APOB* PTV was present in 0.038% of CHD cases as compared to 0.092% of controls. *APOB* PTV carrier status was associated with a 43 mg/dL lower LDL-C ($p=2x10^{-7}$), a 30% decrease in triglycerides ($p=5x10^{-4}$), and a 72% lower risk for CHD (odds ratio=0.28, 95%CI: 0.12-0.64; p=0.002).

Conclusions: Rare PTV mutations in *APOB* which are associated with lower LDL-C and reduced triglycerides also confer protection against CHD.

Key words: genetics, human; genetics, association studies; coronary heart disease; cholesterol

American Heart Association

Circulation: Genomic and Precision Medicine

Introduction

Apolipoprotein B (APOB) is a structural component of lipoproteins with a functional role as a ligand that binds to cell-surface receptors including the low-density lipoprotein (LDL) receptor[1]. Rare protein-truncating variants (PTVs) that truncate *APOB* lead to familial hypobetalipoproteinemia (FHBL, OMIM #107730), an autosomal dominant genetic disorder characterized by low levels of plasma low-density lipoprotein cholesterol (LDL-C) [2, 3]. Those affected by FHBL display not only lower LDL-C but also non-alcoholic fatty liver disease.

Mipomersen is an antisense drug approved by the U.S. Food and Drug Administration that targets the messenger RNA for *APOB* and inhibits the synthesis of the apoB protein. Mipomersen is approved to lower cholesterol in individuals with homozygous familial hypercholesterolemia (HoFH) [4]. Mipomersen leads to a significant decrease in LDL-C levels in individuals with HoFH; however, similar to *APOB* PTVs, mipomersen also leads to fatty liver and elevated liver function test abnormalities[5].

American Heart
Circulation: Genomic and Precision Medicine

Carriers of PTVs in *APOB* display lower LDL-C[6] and triglyceride levels and as such, might be expected to have reduced risk for coronary heart disease (CHD). However, to date, there is little evidence as to whether loss of *APOB* function will affect CHD risk [7, 8] and a pharmacologic test of this hypothesis with mipomersen seems unlikely due to the adverse effects of this therapy. As such, here, we took a human genetics approach to address the following: 1) the extent to which *APOB* PTV carrier status is associated with serum lipid levels using 29 Japanese FHBL families; and 2) whether PTVs in the *APOB* gene are associated with lipid levels and CHD among approximately 58,000 individuals from large case-control studies.

Methods

All participants in the study provided written informed consent for genetic studies. The institutional review boards at the Broad Institute and each participating institution approved the study protocol. In order to minimize the possibility of unintentionally sharing information that can be used to re-identify private information, a subset of the data generated for this study are available at dbGaP and can be accessed at through dbGaP Study Accessions: phs000814.v1.p1 (ATVB), phs001398.v1.p1 (BRAVE), phs000279.v2.p1 (EOMI), phs001098.v1.p1 (JHS), phs001000.v1.p1 (Leicester), phs000990.v1.p1 (NorthGermanMI), phs000916.v1.p1 (SouthGermanMI), phs000806.v1.p1 (OHS), phs000883.v1.p1 (PROCARDIS), phs000917.v1.p1 (PROMIS), phs000902.v1.p1(Regicor).

The full methods are available in the supplemental material.

American Heart Association
Circulation: Genomic and Precision Medicine

Results

Hypobetalipoproteinemia families

In FHBL pedigrees, we tested whether *APOB* PTVs were associated with serum lipids and apolipoproteins. We recruited 29 Japanese FHBL families, and sequenced the exome in 69 participants from the families. Of those, 12 individuals in 4 families and 4 single probands harbored *APOB* PTVs that appeared causative (Supplemental Figure 1). Among these individuals, 3 carried PTVs in homozygous state and 13 harbored PTVs in heterozygous form. Identified causative variants were confirmed through Sanger sequencing (primers shown in Supplemental Table 1). Five of these *APOB* PTVs had not been previously described in FHBL families (Supplemental Table 2). The *APOB* PTVs co-segregated with serum LDL-C and apolipoprotein B levels. Both homozygote and heterozygous carriers exhibited reduction of

serum LDL-C, triglyceride, and apolipoprotein B levels (Figure 1, Supplemental Table 3). Based on linear regression for effect size (95% CI), carrying a PTV in *APOB* was associated with lower LDL-C (-55 mg/dL; 95% CI: -68 to -42; *Mann-Whitney U* p-value = 2.7×10^{-5}), lower triglyceride levels (-53%; 95% CI: -72 to -21; *Mann-Whitney U* p-value = 1.7×10^{-4}), and lower apolipoprotein B (-43 mg/dL; 95% CI: -53 to -33; *Mann-Whitney U* p-value = 2.1×10^{-3}) after adjusting for age and sex.

In the set of Japanese FHBL individuals, *APOB* PTV carriers had higher hepatobiliary enzymes compared to non-carriers (Supplemental Table 3). The three individuals homozygous for APOB PTV were all > 40 years old with evidence of fatty liver on imaging and associated elevation in hepatobiliary enzymes. (Supplemental Table 4).

Association of *APOB* PTVs with lipids and CHD

American Heart Association.
Circulation: Genomic and Precision Medicine

We sequenced the *APOB* gene in a total of 57,973 participants from the Myocardial Infarction Genetics Consortium (MIGen) of African, European, and South Asian ancestries (N=33,835), and from participants of European ancestry (N=24,138) in the Geisinger Health System and Regeneron Genetics Center DiscovEHR study who were recruited as part of the MyCode Community Health Initiative [9] (Table 1). Across a total of 57,973 individuals in 12 studies (Supplemental Table 5), we observed 37 *APOB* PTVs. 32 (86%) of these PTVs were only seen in a single individual (Supplemental Table 6). These mutations included 19 nonsense single-nucleotide substitutions, 3 single-nucleotide substitutions that were predicted to disrupt splicing, and 15 frameshift indels. In aggregate, these 37 mutations were seen in a total of 56 individuals in heterozygous form. No homozygotes or compound heterozygotes were observed.

Among MIGen individuals free of CHD, we found that *APOB* PTV carriers had 43 mg/dL lower LDL-C (95% CI: -59.4 to -26.9; p-value=2.1×10^{-7}), 53 mg/dL lower total

cholesterol (95% CI: -72.4 to -34.3; p-value=$4.2x10^{-8}$), 4 mg/dL higher HDL-C (95% CI: -0.39 to 8.8; p=0.07), and 32% lower triglycerides (95% CI: 15% to 45%; p=$5.0x10^{-4}$) (Table 2). Additionally, among 37,912 individuals in DiscovEHR, *APOB* PTV carriers had a 48 mg/dL lower LDL-C (95% CI: -61.9 to -33.4; p-value=$5.6x10^{-11}$).

Among the 18,442 individuals with CHD, 7 individuals carried a PTV in *APOB* (0.038% carrier frequency) compared to 49 of the 39,531 controls (0.092% carrier frequency) (Figure 2). Carriers of *APOB* PTVs had 72% lower risk of CHD when compared with non-carriers (odds ratio=0.28; 95% CI=0.12-0.64; p-value=0.002). In a sensitivity analysis, we found similar results (odds ratio=0.29; 95% CI=0.12-0.71; p-value=0.006) in the MIGen study after adjusting for sex, PCs of ancestry, and cohort.

American Heart Association.
Circulation: Genomic and Precision Medicine

Discussion

In this study, we assessed whether rare PTVs in *APOB* were associated with lower lipid levels and reduced CHD. Among Japanese FHBL families, we found that carrying an *APOB* PTV in heterozygous form was associated with lower apoB, LDL-C, and triglycerides. Among more than 57,000 participants with and without CHD, *APOB* PTV carrier status also linked to lower total cholesterol, LDL-C, triglycerides, and a 72% lower risk for CHD when compared to non-carriers. These results permit several conclusions.

First, we demonstrate that *APOB* PTVs are a frequent cause of FHBL among the Japanese in this study. By analyzing 29 pedigrees with an extreme LDL-C phenotype, we identified 13 heterozygous carriers and 3 homozygous carriers. Identification of such individuals can enable deep phenotyping to understand the consequences of lifelong perturbation. For example, we note that each of the 3 homozygotes had not only extremely low LDL-C but also

evidence of fatty liver. The presence of fatty liver is consistent with previous reports of adverse effects of using *APOB* inhibitors [10, 11].

Second, we provide evidence that, despite an increased risk of fatty liver, carriers of *APOB* PTVs are at substantially reduced risk of CHD. These findings are of particular importance because clinical trials of mipomersen for CHD outcomes are highly unlikely to be undertaken due to the associated adverse liver effects of mipomersen. These results emphasize the dominant role of apoB containing lipoproteins in protection from CHD.

Third, our results add to a growing body of evidence demonstrating that rare variants associated with reduced circulating apoB-containing lipoproteins are associated with reduced risk of CHD. Rare nonsense mutations in the *PCSK9* gene was noted in 2.6% of blacks and associated with a 88% reduction in risk for CHD[12]. Also, *NPC1L1* rare inactivating variants are observed 1 in 650 individuals and linked to a 53% relative risk reduction for CHD[13].

Strengths of this study include the large sample size and the evaluation of family-based and population-based samples. However, we were not able to assess hepatic enzymes in the population-based samples, we did not functionally validate PTVs, and we were unable to compare effects stratified by ancestry groups given the small number of individuals carrying PTVs within each study.

American Heart Association
Circulation: Genomic and Precision Medicine

Conclusion

Rare PTVs in the *APOB* gene associated with lower LDL-C, lower triglycerides and decreased risk for CHD.

Sources of Funding: GMP is supported by the National Heart, Lung, and Blood Institute of the National Institutes of Health under Award Number K01HL125751. AN was supported by the Yoshida Scholarship Foundation. AK is supported by an institutional grant from the Broad

Institute of MIT and Harvard (BroadIgnite), a K08 from the National Human Genome Research Institute (K08HG010155), and a Junior Faculty Award from the National Lipid Association. SK is supported by a research scholar award from the Massachusetts General Hospital, the Donovan Family Foundation, and grant R01 HL127564 from the National Heart, Lung, and Blood Institute. Funding for the Exome Sequencing Project Early-Onset Myocardial Infarction study was provided by grants RC2 HL103010 (HeartGO), RC2 HL102923 (LungGO), and RC2 HL102924 (WHISP) from the National Heart, Lung, and Blood Institute. Exome sequencing was performed through grants RC2 HL102925 (BroadGO) and RC2 HL102926 (SeattleGO) from the National Heart, Lung, and Blood Institute. Exome sequencing in ATVB, the Precocious Coronary Artery Disease study (PROCARDIS), the Ottawa Heart Study, PROMIS, South German Myocardial Infarction study, and the Jackson Heart Study was supported by grant 5U54HG003067 from the National Institutes of Health (Drs Lander and Gabriel). Field work, genotyping, and standard clinical chemistry assays in PROMIS were principally supported by grants awarded to the University of Cambridge from the British Heart Foundation, U.K. Medical Research Council, Wellcome Trust, EU Framework 6–funded Bloodomics Integrated Project, Pfizer, Novartis, and Merck. Additional support for PROMIS was provided by the U.K. Medical Research Council (MR/L003120/1), British Heart Foundation (RG/13/13/30194), U.K. National Institute for Health Research Cambridge Biomedical Research Centre, European Research Council (268834), and European Commission Framework Programme 7 (HEALTH-F2-2012-279233). The Jackson Heart Study is supported by contracts HHSN268201300046C, HHSN268201300047C, HHSN268201300048C, HHSN268201300049C, and HHSN268201300050C from the National Heart, Lung, and Blood Institute (NHLBI) and the National Institute on Minority Health and Health Disparities. Dr. Wilson is supported by U54GM115428 from the National Institute of General Medical Sciences. REGICOR study was supported by the Spanish Ministry of Economy and Innovation through the Carlos III Health Institute (Red Investigación Cardiovascular RD12/0042, PI09/90506), European Funds for Development (ERDF-FEDER), and by the Catalan Research and Technology Innovation Interdepartmental Commission (2014SGR240). Samples for the Leicester cohort were collected as part of projects funded by the British Heart Foundation (British Heart Foundation Family Heart Study, RG2000010; UK Aneurysm Growth Study, CS/14/2/30841) and the National Institute for Health Research (NIHR Leicester Cardiovascular Biomedical Research Unit

Biomedical Research Informatics Centre for Cardiovascular Science, IS_BRU_0211_20033). The Munich MI Study is supported by the German Federal Ministry of Education and Research (BMBF) in the context of the e:Med program (e:AtheroSysMed) and the FP7 European Union project CVgenes@target (261123). Additional grants were received from the Fondation Leducq (CADgenomics: Understanding Coronary Artery Disease Genes, 12CVD02). This study was also supported through the Deutsche Forschungsgemeinschaft cluster of excellence “Inflammation at Interfaces” and SFB 1123. The Italian Atherosclerosis, Thrombosis, and Vascular Biology (ATVB) Study was supported by a grant from RFPS-2007-3-644382 and Programma di ricerca Regione-Università 2010-2012 Area 1–Strategic Programmes–Regione Emilia-Romagna. The authors would like to thank the MyCode® Community Health Initiative participants for their permission to utilize their health and genomics information in the DiscovEHR collaboration. The DiscovEHR study was funded, in part, by the Regeneron Genetics Center.
The views expressed in this manuscript are those of the authors and do not necessarily represent the views of the NHLBI; the National Institutes of Health; or the U.S. Department of Health and Human Services

American Heart Association.
Circulation: Genomic and Precision Medicine

Disclosures: Dr. Kathiresan reports grant support from Regeneron and Bayer, grant support and personal fees from Aegerion, personal fees from Regeneron Genetics Center, Merck, Celera, Novartis, Bristol-Myers Squibb, Sanofi, AstraZeneca, Alnylam, Color Genomics, Corvidia, Eli Lilly, and Leerink Partners, personal fees and other support from Catabasis, and other support from San Therapeutics outside the submitted work. He holds equity in Verve Therapeutics and Maze Therapeutics. He is also the chair of the scientific advisory board at Genomics plc. Drs. Teslovich, Alex Li, Baras, and Dewey are employees of Regeneron Pharmaceuticals.

References:

1. Davis RA. Cell and molecular biology of the assembly and secretion of apolipoprotein B-containing lipoproteins by the liver. *Biochim Biophys Acta.* 1999;1440:1-31.

2. Schonfeld G. Familial hypobetalipoproteinemia: a review. *J Lipid Res*. 2003;44:878-83.

3. Linton MF, et al. Familial hypobetalipoproteinemia. *J Lipid Res*. 1993;34:521-41.

4. Rader DJ, Kastelein JJ. Lomitapide and mipomersen: two first-in-class drugs for reducing low-density lipoprotein cholesterol in patients with homozygous familial hypercholesterolemia. *Circulation*. 2014;129:1022-32.

5. Raal FJ, et al. Mipomersen, an apolipoprotein B synthesis inhibitor, for lowering of LDL cholesterol concentrations in patients with homozygous familial hypercholesterolaemia: a randomised, double-blind, placebo-controlled trial. *Lancet*. 2010;375:998-1006.

6. Burnett JR, Hooper AJ. Common and rare gene variants affecting plasma LDL cholesterol. *Clin Biochem Rev*. 2008;29:11-26.

7. Welty FK, et al. Identification and molecular analysis of two apoB gene mutations causing low plasma cholesterol levels. *Circulation*. 1995;92:2036-40.

8. Sankatsing RR, et al. Hepatic and cardiovascular consequences of familial hypobetalipoproteinemia. *Arterioscler Thromb Vasc Biol*. 2005;25:1979-84.

9. Dewey FE, et al. Inactivating Variants in ANGPTL4 and Risk of Coronary Artery Disease. *N Engl J Med*. 2016;374:1123-33.

10. Visser ME, et al. Effect of apolipoprotein-B synthesis inhibition on liver triglyceride content in patients with familial hypercholesterolemia. *J Lipid Res*. 2010;51:1057-62.

11. Thomas GS, et al. Mipomersen, an apolipoprotein B synthesis inhibitor, reduces atherogenic lipoproteins in patients with severe hypercholesterolemia at high cardiovascular risk: a randomized, double-blind, placebo-controlled trial. *J Am Coll Cardiol*. 2013;62:2178-84.

12. Cohen JC, et al. Sequence variations in PCSK9, low LDL, and protection against coronary heart disease. *N Engl J Med*. 2006;354:1264-72.

13. Myocardial Infarction Genetics and CARDIoGRAM Exome Consortia Investigators, et al. Inactivating mutations in NPC1L1 and protection from coronary heart disease. *N Engl J Med*. 2014;371:2072-82.

American Heart Association
Circulation: Genomic and Precision Medicine

Table 1. Baseline Characteristics of Myocardial Infarction Genetics Consortium and DiscovEHR Study Participants.

	Myocardial Infarction Genetics Consortium		Geisinger Health System DiscovEHR Cohort	
	CHD	CHD-free	CHD	CHD-free
	Cases	Controls	Cases [‡]	Controls
	N = 14,243	N = 19,592	N = 4,199	N = 19,939
Age, years, mean (SD)	46.2 (8.0)	56.5 (12.1)	51.8 (7.3) [*]	45.0 (12) [*]
Male gender, n (%)	10930 (77)	14556 (74)	1,938 (46)	3,848 (19)
BMI, kg/m^2, median (IQR)	26.8 (24.1–30.1)	26.2 (23.8–29.0)	32.3 (28–38)	31.0 (26–37)
Current smoker, n (%)	6307 (48)	4463 (24)	986 (23)	4,065 (20)
**Ancestry**				
European	6682 (47)	7201 (37)	4,199 (100)	19,939 (100)
Asian	7180 (51)	11045 (57)	0 (0)	0 (0)
African	206 (1)	1128 (6)	0 (0)	0 (0)
Other	28 (<0.001)	0 (0)	0 (0)	0 (0)
**Medical history**				
Hypertension [‖], n (%)	3212 (31)	5548 (36)	3,373 (80)	12444 (34)
Type 2 Diabetes [§], n (%)	1872 (15)	2056 (12)	1,520 (36)	2611 (13)
Lipid-lowering medication [*], n (%)	3463 (35)	538 (4)	2,494 (59)	3639 (18)
**Lipid profile (mg/dL)**				
LDL cholesterol [†], mean (SD)	142 (53.9)	119 (43)	130 (40)	122 (37)
HDL cholesterol, mean (SD)	37 (12)	41 (14)	46 (13)	52 (15)
Triglycerides, median (IQR)	167 (117–247)	151 (102–222)	154 (112–215)	119 (85–167)
Total cholesterol [†], mean (SD)	219 (58)	194 (49)	214 (43)	203 (42)

[*] At the time of median lifetime lipid measurement.

[†] Total and LDL cholesterol values were divided by 0.8 and 0.7 respectively in those on lipid-lowering medication to estimate untreated values.

[‡] Participants were considered to have early-onset (men <55 years, women <65 years) coronary heart disease (CHD) if they had a history of coronary revascularization in the electronic health records, or history of acute coronary syndrome, ischemic heart disease, or exertional angina (ICD-9 codes 410*, 411*, 412*, 413*, 414*) with angiographic evidence of obstructive coronary atherosclerosis (>50% stenosis in at least one major epicardial vessel from catheterization report).

[§] Participants were considered to have diabetes if they had a history of type 2 diabetes in the electronic health records, antidiabetic medication use, or fasting glucose greater than 126 mg/dL or hemoglobin A1c greater than 6.5%.

[‖] Participants were considered to have hypertension if they had a history of hypertension in the electronic health records, antihypertensive medication use, or systolic blood pressure greater than 140 mmHg or diastolic blood pressure greater than 90 mmHg.

SD: standard deviation; IQR: interquartile range.

American Heart Association.
Circulation: Genomic and Precision Medicine

Table 2. Associations of *APOB* protein truncating variant carrier status with plasma lipids in the Myocardial Infarction Genetics Consortium.

**Lipid level**	**N**	**Effect size**	**SE**	**P-value**
LDL cholesterol (mg/dL)	14,754	-43.14	8.30	2.1×10^{-7}
HDL cholesterol (mg/dL)	15,283	4.20	2.34	0.07
Total cholesterol (mg/dL)	15,466	-53.31	9.72	4.2×10^{-8}
Triglycerides (log(mg/dL))	15,787	-0.38	0.11	5.0×10^{-4}

Results are adjusted for PC1-5, cohort, and sex.

American Heart Association.
Circulation: Genomic and Precision Medicine

Figure Legends:

Figure 1. Serum LDL-C (A), triglyceride (B), and apolipoprotein B (C) among Japanese individuals with heterozygous (n=13) and homozygous (n=3) *APOB* protein truncating variant carriers and non-carriers (n=6). Each dot represents an individual's lipid level. Each horizontal line indicates mean value of the lipid level for each genotype. P-values were calculated using *Mann-Whitney U test*. *: P<0.05, **: P<0.01 compared with non-carriers.

PTV, protein truncating variant

Figure 2 (Central Illustration). Association of *APOB* protein truncating variant carrier status with risk of coronary heart disease (CHD) among 57,973 individuals. In each study, the relationship of protein truncating variants in *APOB* with risk of CHD was determined. Exact methods were used to calculate p-values for association tests and confidence intervals (CI). Cochran-Mantel-Haenszel statistics for stratified 2-by-2 tables was performed for meta-analysis. Odds ratio in the Jackson Heart Study (JHS) and North German MI studies were not available due to a lack of observed *APOB* protein truncating variant carriers. ATVB indicates Atherosclerosis, Thrombosis, and Vascular Biology Italian Study; DiscovEHR, DiscovEHR project of the Regeneron Genetics Center and Geisinger Health System; EOMI, Exome Sequencing Project Early-Onset Myocardial Infarction study; Leicester, Leicester Myocardial Infarction study; NorthGermanMI, North German Myocardial Infarction study; OHS, Ottawa Heart Study; PROCARDIS, Precocious Coronary Artery Disease study; PROMIS, Pakistan Risk of Myocardial Infarction Study; REGICOR, Registre Gironí del COR (Gerona Heart Registry) study; SouthGermanMI, South German Myocardial Infarction study.

American Heart Association
Circulation: Genomic and Precision Medicine

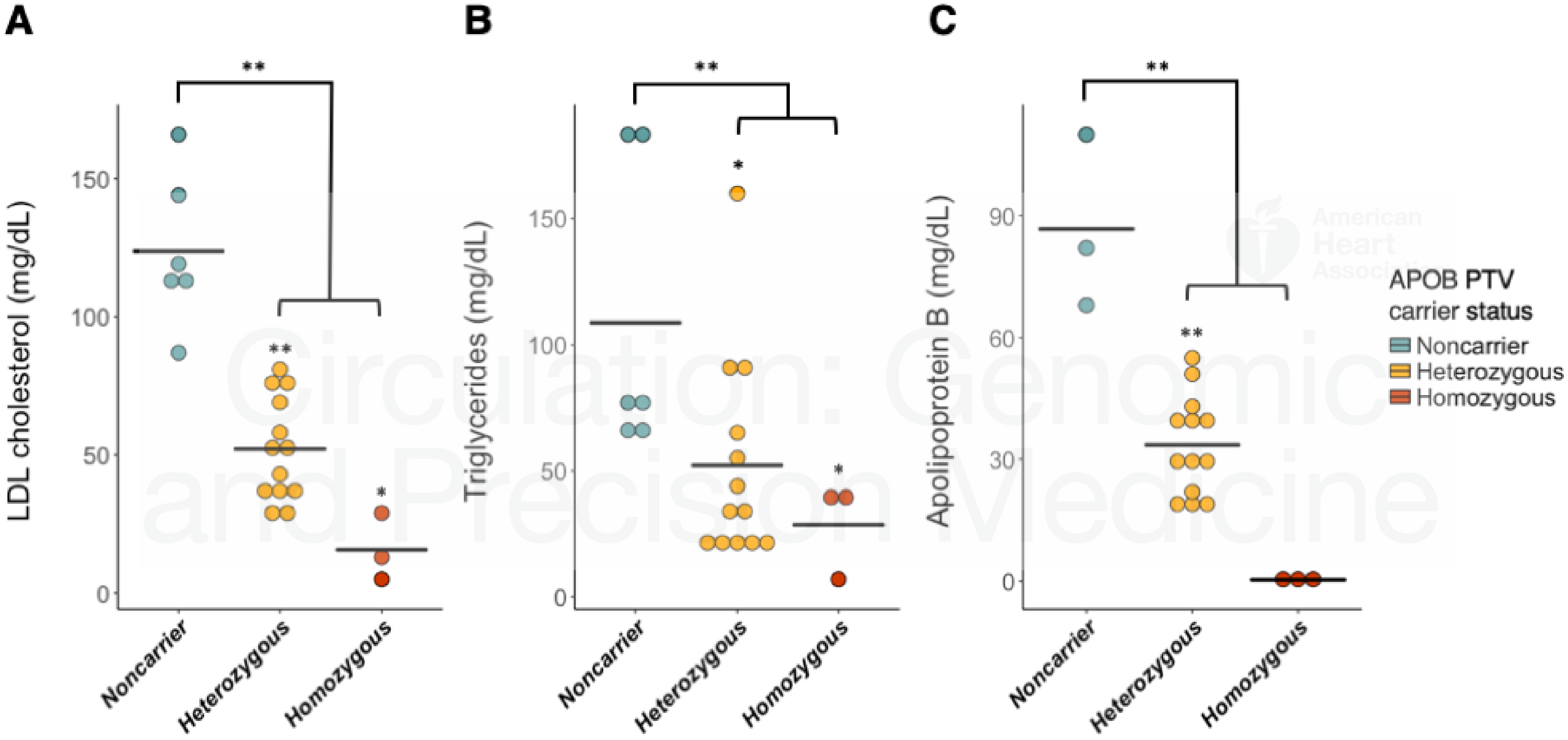

Circulation: Genomic and Precision Medicine
American Heart Association

Study	*APOB* Carriers Case	*APOB* Carriers Cntrl	Noncarriers Case	Noncarriers Cntrl	OR	95%−CI	pval
**MI Genetics Consortium**							
ATVB	1	1	1781	1719	0.97	[0.06; 15.44]	0.980
BRAVE	0	1	745	739	0.33	[0.01; 8.13]	0.498
EOMI	1	0	966	1419	4.41	[0.18; 108.27]	0.364
JHS	0	1	14	706	16.24	[0.63; 415.77]	0.092
Leicester	1	3	1178	1096	0.31	[0.03; 2.99]	0.311
Lubeck	0	0	865	873			
MUNICH	0	0	400	398			
OHS	0	1	575	979	0.57	[0.02; 13.95]	0.729
PROCARDIS	0	2	967	957	0.20	[0.01; 4.13]	0.296
PROMIS	3	20	6380	10283	0.24	[0.07; 0.81]	0.022
REGICOR	0	0	366	394			
**Combined**	**6**	**29**	**14237**	**19563**	**0.29**	**[0.12; 0.72]**	**0.007**
Heterogeneity: I−squared=27.5%, p=0.2087							
**Geisinger Health System**							
DiscovEHR	1	20	4198	19919	0.24	[0.03; 1.77]	0.160
**Overall**	**7**	**49**	**18435**	**39482**	**0.28**	**[0.12; 0.64]**	**0.002**
Heterogeneity: I−squared=19.6%, p=0.2686							

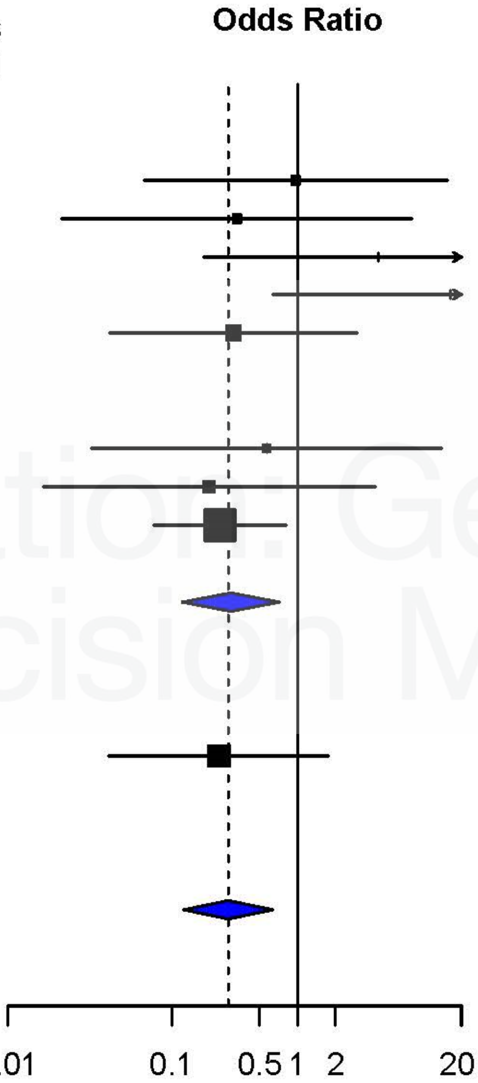

Circulation: Genomic and Precision Medicine
American Heart Association
